# Improving survival in salivary duct cancer with adjuvant androgen deprivation therapy

**DOI:** 10.18632/oncotarget.27004

**Published:** 2019-06-11

**Authors:** Wim van Boxtel, Carla van Herpen

**Affiliations:** Department of Medical Oncology, Radboud University Medical Center, Nijmegen, The Netherlands

**Keywords:** salivar gland neoplasms, antineoplastic agents, hormonal, adjuvant therapy, androgen deprivation therapy

Salivary gland cancer is a rare cancer, with a global annual incidence of 0.4–2.6/100.000 people [1]. Salivary duct carcinoma (SDC) is an aggressive subtype and represents 9% of salivary malignancies [1]. Median overall survival (OS) of SDC patients is 3–5 years after primary diagnosis [2]. In recurrent and/or metastatic SDC, androgen deprivation therapy (ADT) is first line palliative treatment in most patients, as 67–96% of SDCs is androgen receptor-positive (AR+) and an overall response rate of 42% was found in a recently conducted prospective phase 2 trial [3]. We studied as first the added value of adjuvant ADT for poor-risk AR+ SDC patients [4].

**Figure 1:**
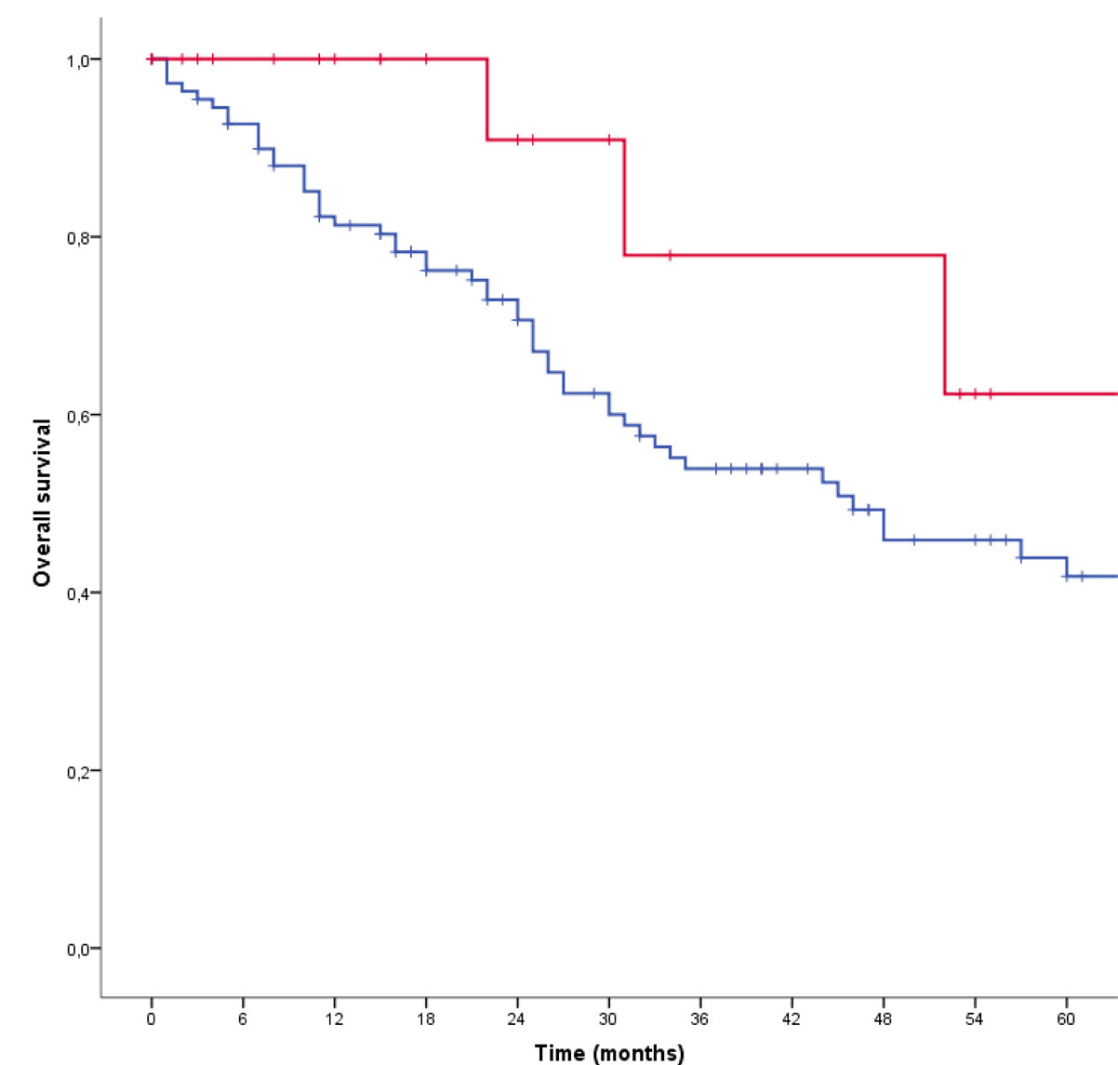
Kaplan-Meier survival curves of the overall survival of adjuvant androgen deprivation therapy-treated patients (red) and the control group (blue) (*P* = 0.074). After correcting for confounders in a multivariable Cox regression analysis, a significant decreased hazard ratio of 0.06 (95% confidence interval 0.01-0.76, *P* = 0.03) was found.

In a retrospective cohort study, 22 patients with stage 4a (T4a and/or N2 and M0) AR+ SDC were treated with adjuvant ADT, i.e. bicalutamide, a luteinizing hormone-releasing hormone (LHRH)-analogue or a combination of both after regular curative treatment. This consisted of a tumor resection in all cases, and a neck dissection and postoperative radiotherapy in most cases. Patients were treated in the Radboud university medical center (Nijmegen, the Netherlands) and the Istituto Nazionale dei Tumori (Milan, Italy). Disease free survival (DFS) and OS data were compared to a control group of 111 stage 4a SDC patients that received regular curative treatment only. After a median follow-up of 20 months in the ADT-treated patients and 26 months in the control group, the 3-year DFS was estimated as 48.2% (95% confidence interval [CI] 14.0–82.4%) and 27.7% (95% CI 18.5–36.9%), respectively (*P* = 0.037). Three-year OS was 77.9% (95% CI 49.7–100%) and 53.9% (95% CI 43.5–64.3%), respectively (*P* = 0.074). See Figure 1.

A univariable and multivariable Cox regression analysis was performed to correct for potential bias. In the univariable analysis, adjuvant ADT treated patients showed a hazard ratio of 0.49 (95% CI 0.21–1.14, *P* = 0.098) for DFS and 0.41 (95% CI 0.13–1.33, *P =* 0.137) for OS. In the multivariable analysis, we corrected for the following potential confounders: gender, age, treatment centre, location of primary tumor, T-stage, N-stage, ex pleomorphic adenoma (yes/no), resection margins, number of positive lymph nodes, postoperative radiotherapy (yes/no), adjuvant chemotherapy (yes/no), AR-status and year of diagnosis. This resulted in a significantly decreased hazard ratio for the adjuvant ADT treated patients of 0.14 (95% CI 0.03–0.75, *P =* 0.022) for DFS and 0.06 (95% CI 0.01–0.76, *P =* 0.030) for OS, compared to the control group.

Several questions remain. Firstly, the optimal treatment regimen is unknown. In our study AR+ SDC patients were treated with bicalutamide monotherapy, a LHRH-analogue, or a combination of both. In prostate cancer, combined androgen blockade shows a modest increase in OS but diminished quality of life [5]. In SDC, no head-to-head comparison has been performed [6, 7]. Novel anti-androgens such as enzalutamide or the CYP17-inhibitor abirateron could be an option for future adjuvant studies. Secondly, the optimal duration of treatment is not obvious. The planned treatment duration in our study differed from 1 to 5 years, but numbers were too small to determine the optimal duration. Finally, the efficacy of ADT in female AR+ SDC patients is of interest. In the palliative setting, there is no reason to presume diminished efficacy, although numbers of treated women is limited. In the adjuvant study, 3 of 22 adjuvant ADT-treated patients were women. They had no evidence of disease after 1, 3 and 14 months of follow-up.

Understanding the mechanisms of primary treatment resistance of ADT in AR+ SDC will be important to predict response and benefit of treatment. Possible mechanisms include low or heterogeneous AR-expression, expression of AR-splice variants such as *AR-V7,* intratumoral androgen synthesis, [8] and interleukin-23 produced by myeloid-derived suppressor cells [9]. Functional inactivity of the AR-pathway, based on interpretation of mRNA expression of AR target genes, is another potential mechanism [10]. Finally, primary treatment resistance may be caused by activity of other tumor-driving pathways, for instance because of *ERBB2* (encoding HER2) amplification or tumor-driving mutations.

Before adopting adjuvant ADT into routine practice for poor-risk SDC patients, our results require confirmation, ideally in a prospective trial. However, due to the rarity of AR+ SDC running a phase III trial is not a real option. Therefore, registration all SDC patients, tumor characteristics, and treatment outcomes in a real-world registry is in our opinion the best way to learn from every patient and thereby improve prognosis of patients rapidly.
